# Doctors' Knowledge of Hypertension Guidelines Recommendations Reflected in Their Practice

**DOI:** 10.1155/2018/8524063

**Published:** 2018-03-12

**Authors:** Nafees Ahmad, Amer Hayat Khan, Irfanullah Khan, Amjad Khan, Muhammad Atif

**Affiliations:** ^1^Faculty of Pharmacy and Health Sciences, University of Balochistan, Balochistan, Pakistan; ^2^Discipline of Clinical Pharmacy, School of Pharmaceutical Sciences, Universiti Sains Malaysia, Pulau Pinang, Malaysia; ^3^Department of Pharmacy, Quaid-e-Azam University, Islamabad, Pakistan; ^4^Department of Pharmacy, The Islamia University, Bahawalpur, Pakistan

## Abstract

**Aim:**

To evaluate doctors' knowledge, attitude, and practices and predictors of adherence to Malaysian hypertension guidelines (CPG 2008).

**Methods:**

Twenty-six doctors involved in hypertension management at Penang General Hospital were enrolled in a cross-sectional study. Doctors' knowledge and attitudes towards guidelines were evaluated through a self-administered questionnaire. Their practices were evaluated by noting their prescriptions written to 520 established hypertensive outpatients (20 prescriptions/doctor). SPSS 17 was used for data analysis.

**Results:**

Nineteen doctors (73.07%) had adequate knowledge of guidelines. Specialists and consultants had significantly better knowledge about guidelines' recommendations. Doctors were positive towards guidelines with mean attitude score of 23.15 ± 1.34 points on a 30-point scale. The median number of guidelines compliant prescriptions was 13 (range 5–20). Statistically significant correlation (*r*_*s*_ = 0.635, *P* < 0.001) was observed between doctors' knowledge and practice scores. A total of 349 (67.1%) prescriptions written were guidelines compliant. In multivariate analysis hypertension clinic (OR = 0.398, *P* = 0.008), left ventricular hypertrophy (OR = 0.091, *P* = 0.001) and heart failure (OR = 1.923, *P* = 0.039) were significantly associated with guidelines adherence.

**Conclusion:**

Doctors' knowledge of guidelines is reflected in their practice. The gap between guidelines recommendations and practice was seen in the pharmacotherapy of uncomplicated hypertension and hypertension with left ventricular hypertrophy, renal disease, and diabetes mellitus.

## 1. Introduction

High prevalence and poor control of hypertension have challenged the public health around the world. Malaysia has an effective and widespread system of healthcare working mainly under Ministry of Health. Infant mortality rate, a yard stick in determining the overall efficiency of healthcare, in 2005 was 10, comparing favorably with the United States and Western Europe. Malaysian healthcare consists of a dual-tiered system: government led and funded public sector and a coexisting private healthcare system. The public sector which provides healthcare services to >65% of the population has the country's best healthcare facilities and equipment but the shortage of doctors in government hospitals is the main drawback [1]. Despite the effective healthcare system, the latest National Health and Morbidity Survey (2015) revealed that situation regarding prevalence (30.3%) and control of hypertension (26.8% to 48.5%) in Malaysia is not different than the global picture [2, 3]. Factors contributing to suboptimal control of hypertension are arbitrarily classified into patients, healthcare providers, and system related factors [4]. In order to improve hypertension control, a large number of hypertension management guidelines have been developed and disseminated worldwide. Despite guidelines' availability, dissemination, and potential to improve hypertension control [5–8], published literature from US [6, 9], Zimbabwe [10], Malaysia [3, 11–13], India [14], South Africa [15], Cyprus [16], Sweden [17], Kuwait [18], Jordan [19], Pakistan [20], and Italy [21] suggests the presence of a wide gap between guidelines recommended and actual clinical practices. According to Cabana et al., barriers limiting adherence to guidelines are classified into three categories:* knowledge related factors*, such as lack of awareness and familiarity,* attitude related factors* such as lack of agreement, lack of outcome expectancy, self-efficacy, and motivation, and* behavior related factors*, such as characteristics of patients, guidelines, and practice [22].

Literature review revealed several weaknesses in previous research regarding evaluation of doctors' adherence to hypertension guidelines. As hypertension occurs in isolation in less than 20% cases and is almost always accompanied by other risk factors [23], addressing comorbidities is an important consideration while measuring doctors' adherence with hypertension guidelines. Some of the studies which had evaluated doctors prescribing practices against the guidelines failed to address comorbidities [24], excluded comorbidities [11, 25], or included only one comorbidity [13, 26], while some failed to define explicit criteria for defining guidelines adherence [24]. The majority of these studies had not conducted the review of patient's medical record to find whether divergence from guidelines was justifiable or not [13, 24, 26, 27]. The studies which had used survey data as a tool for measuring adherence with guidelines had the major limitation of reliance on self-reported practices [17, 19, 21], which are always subject to bias [28]. Doctors' attitudes towards guidelines play a significant role in their implementation in clinical practice. Doctors' intentions to use guidelines can be predicted from their attitudes towards guidelines, which are influenced by many factors, such as their knowledge, past clinical experience, beliefs about guidelines, outcome expectations, peers' opinions, and guidelines characteristics [22]. In order to overcome limitations associated with the above-mentioned studies, we evaluated doctors' subjective (knowledge of guidelines recommendations) as well as objective (actual) prescribing practices, addressed multiple comorbidities, developed explicit criteria for measuring guidelines adherence, conducted detailed review of patients' medical records, and evaluated doctors' attitude towards hypertension guidelines. In addition, we also examined relationship between doctors' knowledge, attitudes, and practices on Malaysian Clinical Practice Guidelines on Management of Hypertension (CPG 2008).

## 2. Methods

This was a cross-sectional study conducted at cardiology, nephrology, diabetic, and hypertension clinics of Penang General Hospital (PGH) Malaysia from October 2010 to April 2011. All the doctors practicing at the four clinics (*n* = 26: 13 at cardiology, 5 at nephrology, and 4 at diabetic and hypertension clinics each) were enrolled in the study. Written consent was taken prior to the beginning of the study. CPG 2008 available at http://www.acadmed.org.my/view_file.cfm?fileid=245 was used as reference.

## 3. Evaluation of Doctors' Knowledge and Attitude on CPG 2008

### 3.1. Tool Development

A self-developed, validated, and reliable questionnaire (in Appendix) was used as a tool for evaluating doctors' knowledge and attitudes on CPG 2008. Content validity of the questionnaire was assessed by a panel of experts composed of a cardiologist, a nephrologist, an endocrinologist, a general physician, and a clinical pharmacist. Construct validity of the tool was established by using key check and item response analysis [29]. Face validity of the questionnaire was established by giving it to a group of 10 participants other than those enrolled in the study [29]. Questionnaire was finalized after a series of discussions with the group. Internal consistency of the knowledge evaluating portion of the tool assessed by using Kuder-Richardson formula 20 (K-R 20) [23] yielded good internal consistency of K-R 20 coefficient = 0.733, while internal consistency of attitude evaluating portion was Cronbach's alpha = 0.808 [29]. To assess the stability of the tool, test-retest correlation was used. Pearson's *r* product moment correlation of 0.885 (*P* < 0.001) and 0.890 (*P* < 0.001) yielded an excellent stability of the knowledge and attitude evaluating portion of the tool, respectively [29].

### 3.2. Tool Administration and Scoring

Questionnaire was administered by the principal investigator (NA). In order to avoid the bias of respondents referring to CPG (2008) for answering the questions, they were requested to fill the questionnaire on spot. The knowledge evaluating portion of the questionnaire consisted of 11 multiple-choice questions. A score of “1” point was credited to each correct answer and “0” to each wrong answer and unanswered question. Adequate knowledge of CPG (2008) was defined as “correctly answering 7 out of 11 questions (>60%). As hypertension cannot be treated properly without correctly diagnosing it, therefore a correct answer regarding hypertension definition according to CPG (2008) was included in these 7 answers” [30]. Questions and correct answers included in the knowledge portion of the questionnaire were derived from recommendations included in CPG 2008.

Attitude evaluation portion, consisting of 6 items, was developed on the basis of extensive literature review. These items were based on a 5-point Likert scale ranging from “Strongly Disagree” to “Strongly Agree” and scored as strongly disagree = 1, disagree = 2, undecided = 3, agree = 4, and strongly agree = 5. Negative items were scored reversely, so that the high score reflects more positive attitude.

## 4. Evaluation of Doctors' Practices

In order to evaluate objective prescribing practices, a total of 520 (20 prescriptions per enrolled doctor) prescriptions written to established hypertensive patients were noted. The inclusion criteria were prescription written to hypertensive outpatients with and without comorbidities and aged > 18 and <80 years. A purpose-developed validated data collection form was used to collect patients' demographic and clinical data. Diagnosis of hypertension and other comorbidities was based on documentation from patients' medical record. Multiple comorbidities were noted and reported as different disease entity; diabetes mellitus, renal disease, stroke, and so on were reported individually. Drugs prescribed to the patients were noted by their generic names. Detailed review of the patients' medical record was conducted. Adverse drug reactions, contraindications, and statement about the inefficacy of a drug, due to which the drug is changed or not prescribed, were noted to find acceptable rationales for nonadherence with guidelines.

Prescription written was considered in compliance with guidelines when

(1) CPG 2008 recommended first-line agent for the particular condition was prescribed,

(2) CPG 2008 recommended first-line agents having no contraindications to their use were prescribed to patients with multiple comorbidities,

(3) CPG 2008 recommended first-line agent for a particular condition was not prescribed because of adverse effects caused by the recommended drug, contraindication to its use, or the drug was changed because of inefficacy.

**Figure 1:**
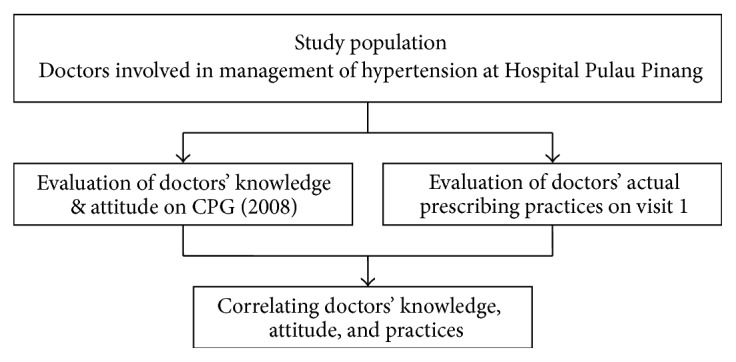
Flowchart of study methodology.

A score of point “1” was credited to each guidelines compliant and “0” to noncompliant prescription. Doctors' knowledge and attitude were correlated to their prescribing practices scores. Flowchart of the study methodology is given in Figure 1.

## 5. Statistical Analysis

Data were analyzed by using SPSS 17. Percentages and frequencies were used for categorical variables, and means, medians, and standard deviations were calculated for continuous variables. Chi-squared and Fisher exact tests were used to observe significance between categorical variables. Mann–Whitney *U* test was performed to observe difference between doctors' demographics and their knowledge, attitude, and practice scores. Univariate logistic regression analysis was conducted to find association between independent variables and CPG adherence. Multivariate analysis was used to obtain a final model describing the significant independent predictors of guidelines adherence. All those variables which had statistically significant association with CPG adherence in univariate analysis were included in multivariate model. The fit of the model was assessed by Hosmer and Lemeshow test and overall classification percentage. Spearman rank order correlation was used to note correlation between doctors' knowledge, attitude, and practice scores. Significance of the statistical tests was taken at a *P* value of <0.05.

This study was approved by the Ministry of Health Medical Research Ethics Committee (MREC), Malaysia (ref: KKM/NIHSEC/08/0804/P-10-453).

## 6. Results

Demographics of the enrolled doctors are given in Table 1. Of the 520 established hypertensive patients included in the final analysis, 304 (58.8%) were males. Mean age of the patients was 61.28 ± 10.98 years. The patients sample was ethnically diverse and consisted of Chinese (259) (49.8%), Malay (168) (32.3%), Indian (81) (15.6%), and other ethnicities (12) (2.3%). A total of 1060 comorbidities were recorded. The most common comorbidity was dyslipidemia (24.62%) (*n* = 261) followed by diabetes mellitus (22.45%) (*n* = 238), IHD (22.45%) (*n* = 238), chronic kidney disease without proteinuria (11.50%) (*n* = 122), HF (8.30%) (*n* = 88), cerebrovascular disease (3.20%) (*n* = 34), asthma (2.35%) (*n* = 25), left ventricular hypertrophy (1.22%) (*n* = 13), gout (1.22%) (*n* = 13), chronic kidney disease with proteinuria (1.13%) (*n* = 12), diabetic nephropathy (1.03%) (*n* = 11), and peripheral vascular disease (PVD) (0.47%) (*n* = 5).

## 7. Doctors' Knowledge and Attitude on CPG (2008)

The percentages of correct answers to the 11 questions are shown in Table 2. The mean number of correct answers was 7.96 ± 1.82 (range 5–11). On the basis of criterion used for adequate awareness, 19 (73.07%) doctors had adequate knowledge of CPG 2008 recommendations. Only three doctors correctly answered all 11 questions. On the basis of designation, we divided doctors into two groups, medical officers and others (specialists and consultants). The results of Mann–Whitney *U* test showed a significant difference (*U* = 17.5, *P* value < 0.001) between knowledge possessed by two groups. Group composed of specialists and consultants was identified to be more knowledgeable (mean rank = 18.25) as compared to medical officers group (mean rank = 7.96).

Doctors in the present study were highly positive towards the CPG (2008), with mean attitude score of 23.15 ± 1.34, ranging from 19 to 24 on a 30-point scale. Doctors' responses to attitude statements are given in Table 3.

## 8. Prescribing Practices

A total of 349 (67.1%) prescriptions were written in compliance with guidelines. The mean number of guidelines compliant prescriptions was 13.42 ± 3.42 ranging from 5 to 20. The results of Mann–Whitney *U* test showed a significant difference (*U* = 31.5, *P* = 0.007) of CPG 2008 adherence score between two groups of doctors. Group composed of specialists and consultants had more guideline adherent practice score (mean rank = 17.25) as compared to medical officers group (mean rank = 9.12).

In univariate analysis, we evaluated association between CPG adherence and patients' age, gender, presence of any comorbidity, HF, LVH, CKD, DM, dyslipidemia, cerebrovascular disease, receiving treatment at cardiology, hypertension, and nephrology clinics. The results of univariate analysis showed that CPG adherence had significant association with comorbidity status, HF, LVH, and treatment at cardiology and hypertension clinics (Table 4). In multivariate analysis, LVH (OR = 0.091, *P* = 0.001) and hypertension clinic (OR = 0.400, *P* = 0.008) had significant negative association, whereas HF had significant positive association (OR = 1.923, *P* = 0.039) with CPG adherence (Table 5). This model fit was based on a nonsignificant Hosmer-Lemeshow test (*P* = 0.975) and overall percentage of 71.1% from the classification table.

Spearman rank correlation yielded a statistically significant strong positive correlation (*r*_*s*_ = 0.635, *P* < 0.001) between doctors' knowledge and practice scores.

## 9. Discussion

Familiarity with guidelines is considered the first step in their implementation in clinical practice [22]. The doctors in the present study possessed comparatively better knowledge than reported by some studies conducted elsewhere. Mean score of correct answers in the present study was 7.96 ± 1.82 points as compared to 5.3 points in a study conducted in Italy [21] and 4.5 in a study conducted in Germany [30]. In comparison to 73.07% of the doctors in the present study, only 23.7% of the German physicians had adequate knowledge about German Society of Hypertension guidelines [30]. However in the present study doctors' knowledge was poor in selecting guidelines recommended antihypertensive agents in LVH, renal disease, and uncomplicated hypertension.

Unfortunately, a very low percentage (23.1%) of doctors in the present study correctly identified the CPG (2008) recommended ARB as preferred antihypertensive therapy for patients with LVH. If we assume that the possible reason for this poor performance could be the nature of the disease, LVH, an advanced and complicated form of cardiovascular disease (CVD), supposed to be treated by cardiologists, still would not justify the doctors' poor performance, because, firstly, all the doctors are supposed to be familiar with guidelines recommendation for treating LVH and secondly half of our respondents (*n* = 13) were practicing in cardiology clinic.

CPG 2008 recommended ACE inhibitors as drug of choice in hypertension with renal disease. Unfortunately, a very low percentage (36.4%) of doctors (*n* = 9) in the present study selected ACE inhibitors as drug of choice in hypertension accompanied by renal disease. A similar low percentage (40.7%) of doctors had correctly answered the same question in a study conducted in Jordan [19].

Because of its association with higher incidence of new onset diabetes mellitus [31] and comparatively lower effectiveness in reducing BP and prevention of stroke [32], use of beta blockers (BB) in uncomplicated hypertension is discouraged by CPG 2008. In the present study, a low proportion (38.5%) of doctors correctly selected BB as antihypertensive agents discouraged in uncomplicated hypertension by CPG 2008. Similar low percentage of doctors had correctly answered the question regarding preferred antihypertensive agents in uncomplicated hypertension in a study conducted in Jordan [19].

All doctors in the present study had welcoming attitudes towards guidelines. They showed trust in both, CPG 2008 and its developers. They believed that CPG 2008 is useful for doctors and adherence to it would produce best patients' outcomes. The reason for doctors welcoming attitudes towards CPG 2008 might be the reputation of the bodies involved in its developing and dissemination. CPG 2008 is developed and disseminated by the Ministry of Health Malaysia in collaboration with Academy of Medicine, Malaysia, and Malaysian Society of Hypertension, the national bodies, which are often perceived to be credible [33]. Beside this, the inclusion of local data and publications in CPG 2008 to ensure local relevance and asking about a specific guideline, that is, CPG 2008, might have played an additional role in doctors' positive attitude towards it. Doctors are found to remain more positive when they are asked about a specific guideline as compared to guidelines in general [34].

Overall, a fair-to-good level of adherence to medication recommendations of CPG 2008 was observed at PGH. More than two-thirds of the patients (67.1%) received guidelines compliant prescriptions. Almost similar findings were reported by a cross-sectional study conducted at a family medicine clinic in Edmonton, where 64% of diabetic or renal disease patients were receiving Canadian Hypertension Education Program (CHEP) recommended therapy [35]. In our study, 67.9% of diabetic and 69% of renal disease patients were receiving CPG 2008 compliant therapy. Adherence to hypertension guidelines in the present study was comparatively better than other studies conducted in Malaysia. A study conducted in two primary clinics has reported that prescribing practices were not in accordance with CPG 2008, as the guidelines discouraged beta blockers were most commonly prescribed drugs in uncomplicated hypertension [11]. Similarly, in another study conducted at 11 primary healthcare clinics in Melaka, state of Malaysia, only 18.3% of diabetic hypertensive patients received guidelines recommended ACE inhibitors as compared to 67.9% in our study [13].

In the present study, doctors' knowledge of CPG 2008 was reflected in their prescribing practices. Doctors remained poorly adhered to those recommendations about which they had poor knowledge. Only 23.1% of the doctors selected guidelines recommended ARB as preferred agents in treating hypertension with LVH. Upon evaluation of the actual prescribing practices, we found only 23.1% of hypertensive patients with LVH were on guidelines compliant therapy. The majority (61.5%) of doctors were unable to correctly identify the guidelines discouraged BB in uncomplicated hypertension. As a consequence, a high percentage (69.4%) of uncomplicated hypertensive patients were on BB. Guidelines recommended BB were selected by almost 77% of the doctors as drug of choice in hypertension with CHD and were prescribed to 77% of patients. However, this relationship between doctors' knowledge and adherence to CPG 2008 medication recommendations did not follow the same sequence in case of diabetes mellitus. More than 86% of doctors selected guidelines recommended ACE inhibitors as drug of first choice in hypertension with diabetes mellitus, but only 64.3% of diabetic hypertensive patients received ACE inhibitors, despite the fact that only 3.6% of patients had contraindications to the use of ACE inhibitors. Similar finding of not implementing the knowledge in clinical practice was observed in studies conducted elsewhere [36, 37].

Hypertension clinic and LVH were the strong predictors of poor adherence to CPG (2008) in both univariate and multivariate analyses. In hypertension clinic, all the four enrolled doctors were medical officers, and in the present study it is observed that medical officers had significantly lower knowledge of CPG 2008 and guideline adherent practice scores as compared to specialists and consultants. Medical officers' inadequate familiarity with CPG 2008 seems to be the reason of poor adherence to guidelines at hypertension clinic. Beside this, all uncomplicated hypertensive patients in the present study were treated in hypertension clinic only. While evaluating doctors' knowledge, we found that the majority of doctors failed in correctly identifying CPG 2008 discouraged BB in uncomplicated hypertension. Prescription of BB to uncomplicated hypertensive patients was the major cause of poor adherence to CPG 2008 in hypertension clinic. Similar guidelines divergent antihypertensive prescriptions in uncomplicated hypertension have also been reported by studies conducted elsewhere. For example, only 18% of the uncomplicated hypertensive patients were on guidelines recommended diuretics in a study conducted elsewhere [25].

As per expectations, specialists and consultants who were more qualified and in practice for longer time performed better than medical officers both in knowledge on CPG 2008 and in practice. This finding is in line with the guidelines adherence models proposed by Cabana et al. [22]. Similarly in studies conducted in Hong Kong and Italy, doctors with higher qualifications and longer duration of practice performed better as compared to doctors with lower qualifications [21, 38]. The other reason for negative association between guidelines adherence and hypertension clinic was comparatively better practices at other clinics, where hypertensive patients with comorbidities were treated. Comparatively better adherence to guidelines at other clinics might be explained by the model proposed by Piette and Kerr [39]. According to the model, patients with concurrent comorbidities of overlapping pathophysiological pathways and management such as hypertension, CVD, and renal disease are likely to receive guidelines adherent management.

In the present study, medical officers at clinics other than hypertension clinic were used to discuss cases with specialists and consultants present at the clinics. In addition to writing guideline adherent prescriptions themselves, the presence of specialists and consultants in these clinics would have definitely helped medical officers in writing guidelines adherent prescriptions. In addition to the medical officers' lower familiarity of CPG (2008), the unavailability of specialists and consultants at hypertension clinic might have an additional effect on poor adherence to guidelines. The reason for poor guidelines adherence in LVH seems to be doctors' lower familiarity of guidelines recommendation as observed while evaluating their knowledge.

Heart failure was the strong predictor of better guidelines adherence in both univariate and multivariate analyses. The possible reason for doctors' good adherence to guidelines while treating patients with heart failure seems to be the wide range of antihypertensive classes (diuretics, ACE inhibitors, BB, ARB, and aldosterone antagonists) recommended by guidelines. The other possible reason for better guidelines adherence might be explained by model proposed by Piette and Kerr [39] which is explained above.

A strong positive relationship was observed between doctors' knowledge score on CPG 2008 and guidelines compliant practice scores. Doctors with higher knowledge of CPG 2008 wrote statistically significant higher numbers of guidelines compliant prescriptions. This finding was in line with the model proposed by Piette and Kerr [39] and studies conducted elsewhere [40, 41]. No significant association was observed among doctors' knowledge and attitude scores and attitude and practice scores. The reason for this lack of relationship seems be the doctors' very positive attitudes towards CPG (2008) irrespective of their knowledge and adherence to it, which made this variable somewhat constant.

## 10. Limitations

Relatively small number of doctors and being an older data set were the major limitations associated with the current study. The reason for doctors limited sample size was the co-relational design of the study. In order to avoid the bias associated with self-reported practices, we evaluated both subjective and objective practices of the doctors, due to which the study was conducted in a single hospital among the small number of doctors who were involved in the management of hypertension. Beside this, both knowledge and practice evaluation sections of the current study focused on hypertension pharmacotherapy, while hypertension management consists of several components including screening, life style interventions, pharmacotherapy, and continued follow-up.

## 11. Conclusion

To the best of our knowledge, this was the first study in Malaysian clinical setup which evaluated both the subjective and objective practices of hypertension management as well as their attitudes towards hypertension management guidelines. More than 73% of doctors were adequately familiar with guidelines recommendations. Lack of knowledge about the particular recommendations was reflected in their practice. Guidelines recommended ARB and ACE inhibitors were underutilized in treating hypertension with LVH and renal disease, while guidelines discouraged BB were prescribed to the patients with uncomplicated hypertension. Medical officers involved in the management of hypertension had significantly lower familiarity of CPG (2008) as compared to specialists and consultants. As lack of knowledge about particular recommendations was the major reason of guidelines divergent practices, multifaceted interventions including education interventions, using reminder tools that indicate appropriate pharmacotherapy, and the availability of clinical pharmacists to participate in collaborative practices which have shown effectiveness in enhancing doctors' adherence to clinical practice guidelines [42, 43] can be used to bridge the gap between evidence based medicines and hypertension management practices at the study site. Medical officers involved in the management of hypertension at (PGH) should be the preferred target population of such interventions. Findings of this study can be used as baseline data and guide for devising suitable interventions to improve doctors' adherence to hypertension guidelines, reduce practice variation, and optimize hypertension control. A large multicenter study including the primary care doctors and using the updated Malaysian CPG (2013) for the management of hypertension as a reference document is recommended to validate the findings of the current study.

## Figures and Tables

**Table 1:** Doctors' demographics.

Variables	Mean ± SD	Number (%)
*Gender*		
Male		10 (38.5)
Female		16 (61.5)
*Age (years)*	35 ± 6.45	
*Years in practice*	8.23 ± 5.11	
*Ethnicity*		
Malay		4 (15.4)
Chinese		17 (65.4)
Indian		4 (15.4)
Others		1 (3.8)
*Designation*		
Medical officers		12 (46.2)
Specialists		6 (23.1)
Consultants		8 (30.8)
*Place of graduation*		
Malaysia		19 (73.1)
Abroad		7 (26.9)

**Table 2:** Percentages of answers conforming to the recommendations of CPG (2008).

Question number	Number and percentage of answers conforming to the guidelines
(1)	24 (92.3)
(2)	22 (84.6)
(3)	22 (84.6)
(4)	16 (61.5)
(5)	10 (48.5)
(6)	22 (84.6)
(7)	24 (92.3)
(8)	17 (64.5)
(9)	6 (23.1)
(10)	9 (34.6)
(11)	20 (76.9)

**Table 3:** Frequencies of doctors' responses of attitude statements towards CPG (2008).

Statement	SA	A	UD	DA	SA
I have trust in the recommendations and developing committee of CPG (2008)	2	24	-	-	-
CPG (2008) on the management of hypertension is helpful for doctors	1	24	1	-	-
Adherence to CPG (2008) would produce desired outcome	1	25	-	-	-
CPG (2008) is motivated by desire to cut cost	-	3	6	17	-
CPG (2008) decreases doctors' autonomy	-	3	1	22	-
CPG (2008) is too rigid to apply to individual patients	-	2	3	21	-

CPG, clinical practice guidelines; SA, strongly agree; A, agree; UD, undecided; DA, disagree; SD, strongly disagree.

**Table 4:** Univariate analysis of predictors of CPG adherence.

Variables	CPG adherence (*n*, %)		Odds ratio	95% CI	*P* value
	Yes	No			
*Gender*					
Male	214 (70.4)	90 (29.6)	Reference		
Female	135 (62.5)	81 (36.5)	0.701	0.484–1.014	0.059
*Age*					
Elderly (≥65 years)	127 (65.5)	67 (34.5)	Reference		
Nonelderly	222 (68.1)	104 (31.9)	1.126	0.773–1.641	0.536
*Comorbidity*					
No	11 (30.6)	25 (69.4)	Reference		
Yes	338 (69.8)	146 (30.2)	5.26	2.52–10.97	0.000
*Heart failure*					
No	277 (64.12)	155 (35.88)	Reference		
Yes	72 (81.81)	16 (18.19)	2.209	1.271–3.841	0.005
*LVH*					
No	346 (68.2)	161 (31.6)	Reference		
Yes	3 (23.1)	10 (76.9)	0.140	0.038–0.514	0.003
*Chronic kidney disease*					
No	256 (66.3)	130 (33.7)	Reference		
Yes	93 (69.4)	41 (30.6)	1.124	0.745–1.698	0.577
*Diabetes mellitus*					
No	188 (66.7)	94 (33.3)	Reference		
Yes	161 (67.6)	77 (32.4)	1.068	0.740–1.541	0.725
*Dyslipidemia*					
No	164 (63.3)	95 (36.7)	Reference		
Yes	185 (70.9)	76 (29.1)	1.410	0.976–2.037	0.067
*Cerebrovascular disease*					
No	323 (66.5)	163 (33.5)	Reference		
Yes	26 (76.5)	8 (23.5)	1.640	0.726–3.703	0.234
*Cardiology clinic*					
No	158 (60.8)	102 (39.8)	Reference		
Yes	191 (73.5)	69 (26.5)	1.79	1.23–2.60	0.002
*Hypertension clinic*					
No	317 (72.0)	123 (28.0)	Reference		
Yes	32 (40.0)	48 (60.0)	0.26	0.16–0.42	0.004
*Nephrology clinic*					
No	279 (66.4)	141 (33.6)	Reference		
Yes	70 (70.0)	30 (30.0)	1.179	0.735–1.893	0.495
*Diabetic clinic*					
No	293 (66.7)	147 (33.3)	Reference		
Yes	56 (70.0)	24 (30.0)	1.171	0.698–1.964	0.551

CI, confidence interval; CPG, clinical practice guidelines; LVH, left ventricular hypertrophy. *Note*. Only statistically significant results are given in the table.

**Table 5:** Multivariate analysis of predictors of CPG adherence.

Variable	B	SE	*P* value	Odds ratio	95% CI
Left ventricular hypertrophy	−2.394	0.688	0.001	0.091	0.024–0.352
Heart failure	0.654	0.318	0.039	1.923	1.032–3.583
Hypertension clinic	−0.915	0.346	0.008	0.400	0.203–0.788

CI, confidence interval; B, beta; SE, standard error. *Note*. Only statistically significant results are given in the table.

## Appendix

### Questionnaire

Please answer the following questions about yourself:

1. Gender
2. □ Male
3. □ Female
4. Age (in years)_________
5. Ethnicity
6. □ Malay
7. □ Chinese
8. □ Indian
9. □ Others, please specify_________
10. Place of graduation
11. □ Malaysia
12. □ Other, please specify_________
13. Designation
14. □ House medical officer
15. □ Medical officer
16. □ Consultant
17. □ Senior Consultant
18. □ Other, please specify_________
19. I am in practice for last_________years.

Pick the single answer for each question that best matches your response.

1. Which of the following blood pressure (BP) values defines hypertension in an adult subject without comorbidities?
2. □ ≥150/90 mm Hg
3. □* ≥140/90 mm Hg*
4. □ ≥135/85 mm Hg
5. □ ≥160/95 mm Hg
6. □ Other please specify_________
7. What is the target BP you would like to achieve in hypertensive patients with* diabetes mellitus and/or chronic kidney disease without proteinuria*?
8. □ <150/95 mm Hg
9. □ <140/90 mm Hg
10. **□*** <130/80 mm Hg*
11. □ <135/85 mm Hg
12. □ Other please specify___________
13. The maximum observational period for a patient recently diagnosed with* Stage 1 Hypertension* having* no evident target organ involvement* and any* additional risk factor is*
14. □ 1 week
15. □ 1 month
16. □ 9 months
17. **□*** 6 months*
18. □ Other please specify__________
19. The absolute risk of cardiovascular events over* 10 years* in* high risk* patients is
20. □ <10%
21. □ 10–20%
22. **□*** 20–30%*
23. □ >30%
24. □ Other please specify____________
25. In newly diagnosed,* uncomplicated hypertension* and no* compelling indications*, all of the following antihypertensive drug classes are agents of* choice of first line* monotherapy except,
26. **□*** Beta blockers*
27. □ Angiotensin Receptor Blockers
28. □ Diuretics
29. □ Calcium Channel Blockers
30. □ Angiotensin Converting Enzymes Inhibitors
31. Which one of the following antihypertensive drug classes you would like to* prescribe* as first choice for hypertensive patients with* diabetes mellitus* having* no proteinuria*?
32. □ Beta blockers
33. □ Calcium Channel Blockers
34. □ Diuretics
35. □ Alpha blockers
36. **□*** Angiotensin Converting Enzymes Inhibitors*
37. Which one of the following antihypertensive drugs you would like to prescribe as first choice for* pregnant hypertensive women*?
38. □* Methyldopa*
39. □ Angiotensin Receptor Blocker
40. □ Diuretics
41. □ Angiotensin Converting Enzyme inhibitor
42. Which one of the following antihypertensive drug classes you would* prefer* for* primary prevention* of stroke?
43. □ Beta Blockers
44. □ Angiotensin Receptor Blockers
45. □* Calcium Channel Blockers*
46. □ Diuretics
47. Which one of the following antihypertensive drug classes you would like to prescribe as* first choice* for hypertensive patient with* left ventricular hypertrophy*?
48. □ Beta Blockers
49. □* Angiotensin Receptor Blockers*
50. □ Calcium Channel Blockers
51. □ Angiotensin Converting Enzyme Inhibitors
52. Which one of the following antihypertensive drug classes you would like to prescribe as* first choice* for hypertensive patients with* non diabetic renal disease*?
53. □ Beta Blockers
54. □ Calcium Channel Blockers
55. □ Alpha Blockers
56. □* Angiotensin Converting Enzyme Inhibitors*
57. Which one of the following antihypertensive drug classes you would like to* prescribe* as* first choice* for hypertensive patients with* coronary heart diseases*?
58. □ Angiotensin Receptor Blockers
59. □* Beta Blockers*
60. □ Alpha Blockers
61. □ Short acting Calcium Channel Blockers

Please circle the response that best describes your beliefs about CPG (2008) on the management of hypertension

1. I have trust in the recommendations and developing committee of CPG (2008)
    Strongly Disagree
    Disagree
    Undecided
    Agree
    Strongly Agree
2. CPG (2008) on the management of hypertension is helpful for doctors
    Strongly Disagree
    Disagree
    Undecided
    Agree
    Strongly Agree
3. Adherence with CPG (2008) would produce desired outcome
    Strongly Disagree
    Disagree
    Undecided
    Agree
    Strongly Agree
4. CPG (2008) is motivated by desire to cut cost
    Strongly Disagree
    Disagree
    Undecided
    Agree
    Strongly Agree
5. CPG (2008) decreases doctors' autonomy
    Strongly Disagree
    Disagree
    Undecided
    Agree
    Strongly Agree
6. CPG (2008) is too rigid to apply to individual patients
    Strongly Disagree
    Disagree
    Undecided
    Agree
    Strongly Agree

## References

[1] Quek D. The Malaysian Health Care System: A Review. https://www.researchgate.net/publication/237409933_The_Malaysian_Health_Care_System_A_Review.

[2] Institute for Public Health (2015). National health and morbidity survey 2015. *Non-Communicable Diseases, Risk Factors and Other Health Problems*.

[3] Teoh S., Razlina A., Norwati D., Siti M. S. (2017). Patients' blood pressure control and doctors' adherence to hypertension clinical practice guideline in managing patients at health clinics in Kuala Muda district, Kedah. *Medical Journal of Malaysia*.

[4] Ahmad N., Hassan Y., Tangiisuran B. (2013). Guidelines adherence and hypertension control at a tertiary hospital in Malaysia. *Journal of Evaluation in Clinical Practice*.

[5] Asmar R., Achouba A., Brunel P., El Feghali R., Denolle T., Vaisse B. (2007). A specific training on hypertension guidelines improves blood pressure control by more than 10% in hypertensive patients: the VALNORM study. *Journal of the American Society of Hypertension*.

[6] Rowan C. G., Turner J. R., Shah A., Spaeder J. A. (2014). Antihypertensive treatment and blood pressure control relative to hypertension treatment guidelines. *Pharmacoepidemiology and Drug Safety*.

[7] Li G., Cai A.-P., Mo Y.-J. (2015). Effects of guideline-based hypertension management in rural areas of Guangdong Province. *Chinese Medical Journal*.

[8] Jackson J. H., Sobolski J., Krienke R., Wong K. S., Frech-Tamas F., Nightengale B. (2008). Blood pressure control and pharmacotherapy patterns in the United States before and after the release of the Joint National Committee on the Prevention, Detection, Evaluation, and Treatment of High Blood Pressure (JNC 7) guidelines. *Journal of the American Board of Family Medicine*.

[9] Levy J., Gerber L. M., Wu X., Mann S. J. (2016). Nonadherence to recommended guidelines for blood pressure measurement. *The Journal of Clinical Hypertension*.

[10] Basopo V., Mujasi P. N. (2017). To what extent do prescribing practices for hypertension in the private sector in Zimbabwe follow the national treatment guidelines? An analysis of insurance medical claims. *Journal of Pharmaceutical Policy and Practice*.

[11] Ramli A. S., Miskan M., Ng K. K. (2010). Prescribing of antihypertensive agents in public primary care clinics - is it in accordance with current evidence. *Malaysian Family Physician*.

[12] Ahmad N., Hassan Y., Tangiisuran B., Meng O. L., Aziz N. A., Khan A. H. (2012). Guidelines adherence and hypertension control in an outpatient cardiology clinic in Malaysia. *Tropical Journal of Pharmaceutical Research*.

[13] Chan G.-C. (2005). Type 2 diabetes mellitus with hypertension at primary healthcare level in Malaysia: are they managed according to guidelines?. *Singapore Medical Journal*.

[14] Raju S., Solomon S., Karthik N., Joseph A. C., Venkatanarayanan (2016). Assessment of prescribing pattern for hypertension and comparison with jnc-8 guidelines-proposed intervention by clinical pharmacist. *Journal of Young Pharmacists*.

[15] Adedeji A. R., Tumbo J., Govender I. (2015). Adherence of doctors to a clinical guideline for hypertension in Bojanala district, North-West Province, South Africa. *African Journal of Primary Health Care and Family Medicine*.

[16] Theodorou M., Stafylas P., Kourlaba G., Kaitelidou D., Maniadakis N., Papademetriou V. (2012). Physicians' perceptions and adherence to guidelines for the management of hypertension: a national, multicentre, prospective study. *International Journal of Hypertension*.

[17] Midlöv P., Ekesbo R., Johansson L. (2008). Barriers to adherence to hypertension guidelines among GPs in southern Sweden: a survey. *Scandinavian Journal of Primary Health Care*.

[18] Al-Ali K. A., Al-Ghanim F. A., Al-Furaih A. M., Al-Otaibi N., Makboul G., El-Shazly M. K. (2013). Awareness of hypertension guidelines among family physicians in primary health care. *Alexandria Journal of Medicine*.

[19] Al-Azzam S. I., Najjar R. B., Khader Y. S. (2007). Awareness of physicians in Jordan about the treatment of high blood pressure according to the seventh report of the Joint National Committee (JNC VII). *European Journal of Cardiovascular Nursing*.

[20] Jafar T. H., Jessani S., Jafary F. H. (2005). General practitioners' approach to hypertension in urban Pakistan: disturbing trends in practice. *Circulation*.

[21] Cuspidi C., Michev I., Mean S. (2003). Awareness of hypertension guidelines in primary care: results of a regionwide survey in Italy. *Journal of Human Hypertension*.

[22] Cabana M. D., Rand C. S., Powe N. R. (1999). Why don't physicians follow clinical practice guidelines? A framework for improvement. *Journal of the American Medical Association*.

[23] Long A. N., Dagogo-Jack S. (2011). Comorbidities of diabetes and hypertension: mechanisms and approach to target organ protection. *The Journal of Clinical Hypertension*.

[24] Siegel D., Lopez J. (1997). Trends in antihypertensive drug use in the United States: do the JNC V recommendations affect prescribing?. *Journal of the American Medical Association*.

[25] Drawz P. E., Bocirnea C., Greer K. B., Kim J., Rader F., Murray P. (2009). Hypertension guideline adherence among nursing home patients. *Journal of General Internal Medicine*.

[26] Russell C., Dunbar P., Salisbury S., Sketris I., Kephart G. (2005). Hypertension control: Results from the Diabetes Care Program of Nova Scotia registry and impact of changing clinical practice guidelines. *Cardiovascular Diabetology*.

[27] Hyman D. J., Pavlik V. N. (2000). Self-reported hypertension treatment practices among primary care physicians: blood pressure thresholds, drug choices, and the role of guidelines and evidence-based medicine. *JAMA Internal Medicine*.

[28] Adams A. S., Soumerai S. B., Lomas J., Ross-Degnan D. (1999). Evidence of self-report bias in assessing adherence to guidelines. *International Journal for Quality in Health Care*.

[29] Considine J., Botti M., Thomas S. (2005). Design, format, validity and reliability of multiple choice questions for use in nursing research and education. *Journal of the Royal College of Nursing Australia*.

[30] Hagemeister J., Schneider C. A., Barabas S. (2001). Hypertension guidelines and their limitations - The impact of physicians' compliance as evaluated by guideline awareness. *Journal of Hypertension*.

[31] Elliott W. J., Meyer P. M. (2007). Incident diabetes in clinical trials of antihypertensive drugs: a network meta-analysis. *The Lancet*.

[32] Lindholm L. H., Carlberg B., Samuelsson O. (2005). Should *β* blockers remain first choice in the treatment of primary hypertension? A meta-analysis. *The Lancet*.

[33] Rashidian A., Eccles M. P., Russell I. (2008). Falling on stony ground? A qualitative study of implementation of clinical guidelines' prescribing recommendations in primary care. *Health Policy*.

[34] Olesen F., Lauritzen T. (1997). Do general practitioners want guidelines?: attitudes toward a county- based and a national college-based approach. *Scandinavian Journal of Primary Health Care*.

[35] Houlihan S. J., Simpson S. H., Cave A. J. (2009). Hypertension treatment and control rates: chart review in an academic family medicine clinic. *Canadian Family Physician*.

[36] Oliveria S. A., Lapuerta P., McCarthy B. D., L'Italien G. J., Berlowitz D. R., Asch S. M. (2002). Physician-related barriers to the effective management of uncontrolled hypertension. *JAMA Internal Medicine*.

[37] Holland N., Segraves D., Nnadi V. O., Belletti D. A., Wogen J., Arcona S. (2008). Identifying barriers to hypertension care: implications for quality improvement initiatives. *Population Health Management*.

[38] Wae T., Man C., Tong L., Wan C., Yuk C., Street Y. (2006). Are we evidence-based in prescribing for hypertension?. *Asia Pacific Journal of Family Medicine*.

[39] Piette J. D., Kerr E. A. (2006). The impact of comorbid chronic conditions on diabetes care. *Diabetes Care*.

[40] El-Solh A. A., Alhajhusain A., Saliba R. G., Drinka P. (2010). Physicians' attitudes toward guidelines for the treatment of hospitalized nursing home-acquired pneumonia. *Journal of the American Medical Directors Association*.

[41] Ikeda N., Hasegawa T., Hasegawa T., Saito I., Saruta T. (2006). Awareness of the Japanese Society of Hypertension Guidelines for the Management of Hypertension (JSH 2000) and compliance to its recommendations: surveys in 2000 and 2004. *Journal of Human Hypertension*.

[42] Berwanger O., Guimarães H. P., Laranjeira L. N. (2012). Effect of a multifaceted intervention on use of evidence-based therapies in patients with acute coronary syndromes in Brazil: the BRIDGE-ACS randomized trial. *Journal of the American Medical Association*.

[43] Squires J. E., Sullivan K., Eccles M. P., Worswick J., Grimshaw J. M. (2014). Are multifaceted interventions more effective than single-component interventions in changing health-care professionals' behaviours? An overview of systematic reviews. *Implementation Science*.

